# DAS steered therapy in clinical practice; cross-sectional results from the METEOR database

**DOI:** 10.1186/s12891-016-0878-1

**Published:** 2016-01-16

**Authors:** Emilia Gvozdenović, Ron Wolterbeek, Désirée van der Heijde, Tom Huizinga, Cornelia Allaart, Robert Landewé

**Affiliations:** Department of Rheumatology, Leiden University Medical Center, Albinusdreef 2, 2300 RC Leiden, The Netherlands; Department of Biostatistics, Leiden University Medical Center, Leiden, Netherlands; Academic Medical Center, Amsterdam & Atrium Medical Center, Heerlen, Netherlands

**Keywords:** Rheumatoid arthritis, DMARD therapy, Clinical outcomes

## Abstract

**Background:**

Little is known on how well targeted treatment, for instance targeting towards low DAS, is implemented in clinical practice. Our aim was to evaluate treatment adjustments in response to DAS in RA patients in clinical practice.

**Methods:**

We used data from one referral centre, multiple rheumatologists, from the METEOR database. Generalized Estimating Equations (GEE) were used to assess whether in case of non-low disease activity (DAS > 2.4) treatment intensifications in DMARD therapy occurred ((change or increase in dose or number of DMARDs, including synthetic (s)DMARDs, biologic (b)DMARDs and corticosteroids compared to the visit before)). Determinants of not intensifying the treatment when DAS > 2.4 were investigated using GEE.

**Results:**

Five thousand one hundred fifty-seven registered visits of 1202 patients were available for the analyses. A DAS > 2.4 was weakly (OR: 1.19; 95 % CI 1.07–1.33) associated with a treatment intensification. In 69 % (n = 3577) of the visits patients were in low disease activity. In 66 % (n = 1028) of the visits with DAS > 2.4 treatment was not intensified. These patients had a higher tender joint count and received more often methotrexate plus a bDMARD, or csDMARD monotherapy, as compared to patients that received treatment intensification.

**Conclusion:**

In the majority of visits in the METEOR database patients were already in a state of low disease activity, reflecting appropriate treatment intensity. When DAS was greater than 2.4, treatment was often not intensified due to high tender joint count or specific treatment combinations. This data suggest that while aiming for low DAS, physicians per patient weigh whether all DAS elements indicate disease activity or will respond to DMARD adjustment or not, and make treatment decisions accordingly.

**Electronic supplementary material:**

The online version of this article (doi:10.1186/s12891-016-0878-1) contains supplementary material, which is available to authorized users.

## Background

The aim of treatment in rheumatoid arthritis (RA) is to achieve low disease activity or remission using a ‘treat to target’ (tight control) approach in which the disease activity of patients is monitored intensively and measured frequently with composite measures [1–3]. Treatment intensity can be adjusted by changing DMARDs or by increasing the dose and/or number of anti-rheumatic drugs, including synthetic (s)DMARDs and biologic (b)DMARDs and corticosteroid [4]. Since treatment to target and tight control have been proven to result in better clinical and radiological outcomes than routine care, [5–11] these concepts are at the basis of the current recommendations for the management of rheumatoid arthritis in daily practice. When sustained remission or low disease activity is achieved and medication is tapered or discontinued and following patients and tight control is important as half of the patient may flare with decreasing medication [12, 13].

Despite the fact that rheumatologists have reported to use treat-to-target in daily practice, [14–16] some studies have suggested that that targeted treatment may not be widely practiced yet [17, 18]. Besides, it is well known that limited adherence to guidelines is prevalent in many chronic conditions, such as atrial fibrillation, hypertension and osteoporosis [19–21].

We used the Measurement of Efficacy of Treatment in the ‘Era of Outcome’ in Rheumatology (METEOR) database, [22] to investigate the association between level of the DAS and whether or not physicians adjusted treatment with sDMARDs and bDMARDs and corticosteroids in patients with RA in daily clinical practice.

## Methods

### Patients

For the current cross-sectional analyses we have used data from METEOR, which is an international prospective database aiming to improve tight monitoring and treatment to target in patients with rheumatic diseases. METEOR started in 2008 and is used as an online daily practice tool for rheumatologists to collect clinical data and calculate disease activity, registering the effectiveness of their treatment practice over time in patients with RA. Data of both patients with advanced disease and with newly diagnosed RA were collected in a central database. Data is uploaded anonymously and therefore an ethics statement is not required for this study. A more detailed description of the METEOR database was published previously [22, 23]. Within METEOR we made a sub selection of data from the Leiden University Medical Center (LUMC), since at the time of evaluation the data of this site were most complete with information on both DAS outcomes and anti-rheumatic treatment. LUMC patients were, included in METEOR between January 2008 and May 2013. Data were made available after written application, by permission of the scientific committee of METEOR. We have selected 1202 patients (5157 visits ranging from 1 to 31 visits per patient) where DAS (5157 visits) as well as information on treatment were available.

### Outcome variables and determinants

Treatment adjustment was divided into three categories; 1) dose decrease (either a lower dose or fewer sDMARDs or bDMARDs or corticosteroids, including intra articular injections, compared to the previous visit), 2) stable dose (the same sDMARDs or bDMARDs or corticosteroids, including intra articular injections, and the same dose compared to the previous visit) and 3) treatment change or intensification (higher dose or more or other sDMARDs or bDMARDS or corticosteroids, including intra articular injections, compared to the previous visit). DAS was classified in four categories, according to the EULAR classification criteria (DAS < 1.6 representing clinical remission, DAS > 1.6 and ≤2.4 representing low disease activity, DAS >2.4 and ≤3.7 representing moderate disease activity, and DAS > 3.7 representing high disease activity).

For secondary analyses DAS was divided in two categories; DAS ≤2.4 and DAS > 2.4.

We used hypothetical conditions, based on a previous study, in which there was a discrepancy between components of the DAS representing inflammation (joint swelling, laboratory results) or pain (potentially regardless of inflammation) as secondary outcomes [24]. These conditions included 1) cases in which a patient had ≤1 swollen joints but two or more tender joints 2) cases in which a patient had ≤1 swollen joints but reported a high disease activity (≥20 on a visual analogue scale, VASpt) 3) cases in which a patients had ≤ 1 swollen joints but an erythrocyte sedimentation rate (ESR) ≥ 28 mm/h 4) cases in which VASpt was ≥20 mm higher than the physician’s score of the patients global disease activity (VASphys) and 5) cases in which the VASphys was ≥20 mm higher than the VASpt [23, 25].

### Statistical analyses

Descriptive statistics were performed using median and interquartile ranges (IQR) for continuous variables, and number and percentages for categorical variables.

The association between DAS and treatment adjustments was assessed using generalized estimating equations (GEE) in order to adjust for the spurious effects of repeated measurements and treatment adjustments within the same subject. The probability of a treatment outcome (decreased, stable or change/intensified) was modelled using the GEE ordinal (cumulative) regression analysis approach [26]. In the regression model, treatment adjustment was the dependent variable, with increase of treatment being the last ordinal category (reference). DAS > 2.4 (yes or no) was used as the determinant. We tested the proportional odds for treatment adjustment with crude calculations in cross tables and the goodness-of-fit test. These did not indicate a violation of the proportional odds assumption [27]. In a set of subanalyses, a first GEE binary logistic regression was performed to compare decreased dose versus stable dose. A second binary GEE was performed to compare decreased dose versus increased dose; and a third binary GEE analysis was performed to compare increased dose versus stable dose. In all three analyses DAS > 2.4 was the determinant. A fourth GEE was performed to model patients with DAS > 2.4 (no intensification versus intensification of treatment) as dependent variable, with erythrocyte sedimentation rate (ESR), SJC, VASpt (patient assessment of global disease activity), TJC and actual treatment as determinants. This model was corrected for gender and age.

SPSS version 17.0 was used for the analyses and a two-sided P-value less than 0.05 was considered statistically significant.

## Results

In total 5157 registered visits in 1202 patients who were treated with sDMARDs and/or bDMARDs and/or corticosteroids, were available for the analyses. Mean age was 56 (SD: 14) and disease duration was on average 17 months (IQR: 3–84). 71 % (n = 854) of the population were women (see Additional file 1: baseline characteristics (visit 1) for patients in the METEOR database).

In 1580 of these 5157 visits (31 %) DAS was >2.4 (and in 4 % of these, DAS was >3.7), in 3577 visits (69 %), DAS was ≤2.4 (in 39 % of these DAS was <1.6). In 1692/5157 visits (33 %) medication was intensified, in 2881 visits (56 %), medication was kept stable, and in 584 visits (11 %) medication was tapered or discontinued (Table 1). GEE showed that on patient level a higher DAS was only weakly but yet statistically significantly correlated with a change/increase in medication (OR: 1.19, 95 % CI: 1.07 to 1.33, Table 2). The binary logistic GEE regression showed that in patients with a DAS > 2.4 treatment was more often changed/intensified (OR: 1.30, 95 % CI: 1.05 to 1.60) than tapered, but not significantly more often changed/intensified than kept stable (Table 2). In only 552/1580 (35 %) of the visits in which the patient had a DAS > 2.4 medication was indeed changed/intensified, and this percentage was not higher in patients with a DAS > 3.7 (Table 1). In 23 visits (11 %) in which patients had high disease activity (DAS > 3.7) the dose was even decreased. In comparison, in 629 of the 2035 visits (31 %) where patients were in remission (DAS < 1.6) medication was still changed/intensified (Table 1).

**Table 1:** Number of visits with decreased, stable or increased dose per level of disease activity based on DAS score

		Decreased dose	Stable dose	Increased dose	Total
DAS	Remission: < 1.6, N (%)	257 (13)	1149 (56)	629 (31)	2035 (100)
	^a^LDA: 1.6–2.4, N (%)	179 (12)	852 (55)	511 (33)	1542 (100)
	^a^MDA: 2.4–3.7, N (%)	125 (9)	770 (56)	479 (35)	1374 (100)
	^a^HDA: > 3.7, N (%)	23 (11)	110 (53)	73 (35)	206 (100)

^a^ *LDA* Low Disease Activity, *MDA* Moderate Disease Activity, *HDA* High Disease Activity

**Table 2:** Association between DAS and treatment adjustment in METEOR

	DAS > 2.4^a^	
	ß	OR (95 % CI)
Overall ordinal correlation^b^	0.175	1.19 (1.07–1.33)
Stable versus decreased dose^c^	0.259	1.30 (1.05–1.60)
Increased versus stable dose^c^	0.096	1.10 (0.97–1.26)
Increased versus decreased dose^c^	0.36	1.43 (1.17–1.74)

^a^Reference category is a DAS ≤ than 2.4

^b^Ordinal; GEE (regression analysis). DAS > 2.4 gives higher odds to increase treatment

^c^Binary; GEE (regression analysis)

In the 1028/1580 (65 %) visits in which patients had a DAS > 2.4 treatment was nevertheless not changed/intensified. On the visit level we investigated whether there were discrepancies in DAS components in these 1028 visits by comparing the median (interquartile range, IQR) tender joint count, swollen joint count, ESR and patient VAS: the median for tender joint count (6, IQR 2–8) was slightly higher than the median for swollen joint count (median 4, IQR 2–8) (Table 3). In 20 % of the visits in which DAS was >2.4 and medication was not changed/intensified, SJC was low (≤1) while TJC and patient’s VAS for global disease activity were high (≥2 or ≥20, respectively). A higher patient-reported- than physician-assessed global disease activity (difference in VAS ≥20 mm) was found in 33 % of these visits (Table 4).

**Table 3:** Median of DAS components in visits where medication is not increased when patients have moderate/high disease activity

	DAS > 2.4 and medication not increased
	N = 1028 visits Median (IQR)
VASpt^a^	60.0 (46.0–72.8)
SJC^a^	4.0 (2.0–8.0)
TJC^a^	6.0 (4.0–8.0)
ESR^a^	25.0 (11.0–38.0)^b^

^a^ *ESR* erythrocyte sedimentation rate, *VASpt* Patient assessment of Global Disease Activity, *TJC* Tender Joint Count, *SJC* Swollen Joint Count

^b^ *n* = 1 missing visit for ESR

**Table 4:** Number of visits in which there are discrepancies in DAS components in patients with moderate/high disease activity that did not receive treatment intensification

	DAS > 2.4 and medication not increased	
		Total
SJC ≤1 and TJC ≥2, N (%)	201 (19.6)	1028
SJC ≤1 and VASpt ≥20, N (%)	198 (19.3)	1028
SJC ≤1 and ESR ≥28, N (%)	98 (9.5)	1027
VASpt ≥20 mm higher than VASphys N (%)	148 (32.9)	450
VASphys ≥ 20 mm higher than VASpt, N (%)	25 (5.6)	450

*ESR erythrocyte sedimentation rate, *VASpt* Patient assessment of Global Disease Activity, *TJC* Tender Joint Count, *SJC* Swollen Joint Count, *VASphys* Physician Assessment of Global Disease Activity

In a GEE binary logistic regression we checked in patients with a DAS > 2.4 which factors were associated with NO intensification of treatment (intensification of treatment = reference), corrected for age and gender. These factors were (high) tender joint count (OR: 1.05, 95 % CI: 1.01 to 1.10), current treatment with conventional synthetic (cs) DMARD monotherapy (OR: 3.28, 95 % CI: 2.40 to 4.48) and combination therapy with methotrexate (MTX) and a bDMARD (OR: 1.93, 95 % CI: 1.25 to 2.98) (Table 5).

**Table 5:** Determinants for not increasing medication when patients have moderate/high disease activity

	DAS > 2.4: medication is not increased vs medication is increased (n = 1.574 visits)^a^		
	ß	OR (95 % CI)	*P*-value
ESR^a^	0.00	1.00 (0.99–1.01)	0.86
SJC^a^	−0.02	0.99 (0.96–1.01)	0.20
VASpt^a^	0.00	1.00 (0.99–1.01)	0.98
TJC^a^	0.05	1.05 (1.01–1.10)	<0.01
Actual drug^a^			
DMARD monotherapy	1.19	3.28 (2.40–4.48)	<0.01
MTX + bDMARD	0.66	1.93 (1.25–2.98)	<0.01
DMARD combination therapy	0.12	1.12 (0.83–1.53)	0.46
DMARD + prednisone	0.03	1.03 (0.67–1.57)	0.90

^a^GEE binary logistic regression. Analysis is corrected for gender and age. *ESR* Erythrocyte Sedimentation Rate, *SJC* swollen joint count, *TJC* tender joint count, *VasPtGlobal* Patient Assessment of Global Disease Activity. Reference category ‘Actual drugs’ = other drugs

Finally, we checked on a visit level whether an improvement in DAS was found compared to the previous visit, but also at the following visits. However, we did not have drug data on all the previous and following visits. In 82 of the available 874 visits (9 %) there had been an improvement in DAS (EULAR (European League Against Rheumatism) response moderate or good)) compared to the previous visit (Table 6). After a high DAS was followed with no change or increase in medication, at the following visit a good or moderate improvement in DAS was observed in 47 (17 %) of the recorded 283 visits.

**Table 6:** Number of visits in which patients show improvement in DAS according to the EULAR criteria

	^a^DAS > 2.4, medication	
	Not increased	Total
DAS improvement compared to the previous visit		
None: ≤0.6, N (%)	792 (91)	874
Moderate: 0.6–1.2, N (%)	60 (7)	874
Good: >1.2, N (%)	22 (2)	874
DAS improved in the following visit		
None: ≤0.6, N (%)	236 (83)	283
Moderate: 0.6–1.2, N (%)	33 (12)	283
Good: >1.2, N (%)	14 (5)	283

^*a*^ *D AS* Disease Activity Score

## Discussion

In this analysis from daily practice observations collected in the METEOR database, we obtained information about how the treat to target recommendation in RA is followed in a single large academic referral centre (LUMC). Most patients had low disease activity or remission (69 % of visits DAS = <2.4, 39 % even <1.6) during the majority of visits. These percentages approach figures that have been reported in treat-to-target studies such as CAMERA, [28] DREAM [29] and BeSt [30] in which 50–82 % achieved low disease activity or remission.

This observation, together with the apperception that DAS-results were indeed measured and recorded, support a conclusion that rheumatologists in the LUMC follow the treat to target approach in daily practice quite well. Since rheumatologists working in the LUMC conducted the Best study, which aims at low disease activity using DAS-steered therapy, this could be expected. Many previous studies, such as TICORA, GUEPARD and ESPOIR showed that a treat to target strategy leads to better clinical outcomes compared to routine care [5, 8].

Since questionnaire-based studies suggest that rheumatologists are aware of the advantages of treatment to target and are willing to apply the treatment recommendations, [14–16] the METEOR tool was developed to help and stimulate rheumatologists to apply a treat to target approach in daily practice.

In spite of a high percentage of patients with DAS < 2.4, we also found on a patient level, that DAS > 2.4 itself was only weakly associated with a change or intensification of antirheumatic medication (OR: 1.19). In comparison to tapering the dose if DAS was ≤2.4, the likelihood of increasing the dose was only marginally higher in patients with a DAS >2.4. Furthermore, per visit where treatment was not intensified although DAS was higher than 2.4 we found discrepancies in subjective patient outcomes (high tender joint count and/or high patient reported global disease activity on a visual analogue scale) versus physician assessment of disease activity (low swollen joint count). This is reflected by an OR for tender joint count of 1.05 (CI95Q% 1.01–1.1) for not intensifying medication in case of DAS > 2.4.

This observation may suggest that although rheumatologist may steer treatment decisions by the measured DAS, they consider other explanations of high DAS components, for instance secondary fibromyalgia or irreversible joint damage as explanation for a high tender joint count, which may not respond to a further increase of anti-inflammatory drugs. A discrepancy between subjective patient outcomes and objective physician assessments has been shown in earlier METEOR studies focused on patient’s global disease activity (GDA). Here we found that when patients rate their GDA, they base their opinion more on subjective signs (patient’s perception of pain), while physicians put more weight on objective signs (swollen joint count, ESR) when rating GDA of the patient. Also is shown that discrepancies between patients and physicians in GDA assessment are different among countries, suggesting that reporting and acknowledging pain differs per country [31, 32]. The METEOR database does not contain information on damage or secondary pain syndromes, or indeed other comorbidities, which may also have held rheumatologists back in increasing treatment where the DAS was high. Nor do we have information on reasons why patients may not have wanted to increase medication.

We also found that the likelihood of treatment intensification in case of DAS > 2.4 was less if the patient was currently using csDMARD monotherapy, which may indicate a reluctance among patients to change or expand medication, [33] or methotrexate in combination with a biological agent. The latter may indicate that rheumatologists may be reluctant to change the biologic, as it is currently unclear which is the optimal treatment choice if the first biologic is ineffective [34–36]. Previous studies suggest that an important reason for the rheumatologist to not (yet) intensify the treatment was that they anticipated further improvement on the current medication. We tested this hypothesis but we found only in 9 % of the available visits clinical relevant improvement in DAS.

An important limitation to this study is that we do not have data on comorbidities, which might influence the decision of the rheumatologist to change or not change the treatment. Furthermore, we used a rather broad categorization of treatment adjustment without any hierarchy in for instance type or number of drugs that were adjusted, which may have influenced the results. Another limitation is that we have no imaging data, although presence or absence of radiologic damage progression, in clinical practice can influence the decision on treatment intensification. A final restriction is that we used only data form the LUMC since data of other centers/countries were not (sufficiently) available yet. These results may therefore not be generalizable to all patients treated in clinical practice, since perception of pain seems to be country dependent.

## Conclusions

In conclusion, we have found a high percentage of patients with remission or a low level of disease activity in the majority of regular registered visits of RA patients to the outpatient clinic of a large academic hospital in the Netherlands. We have also found that a moderate- to high disease activity does not automatically lead to treatment intensification, which may still suggest that Treat-to-Target and EULAR recommendations for the management of patients with RA are well followed, but also that the doctor is looking critically at possible reasons for elevation of elements of the DAS before deciding on treatment intensification. Future research is needed to study the relationship between disease activity and treatment adjustment using different categorizations, such as type of medication. Also, it will be useful to understand how comorbidities influence the relationship between treatment adjustment and disease activity.

## Additional file

Additional file 1:
**Baseline characteristics (visit 1) for patients in the METEOR database.** (DOCX 17 kb)

## Abbreviations

RA: Rheumatoid arthritis
DAS: Disease Activity Score
DAS28: DAS28, CDAI and SDAI
DMARDs: Disease-modifying anti rheumatic drug
sDMARDs: Synthetic disease-modifying anti rheumatic drug
bDMARDs: Biologic disease-modifying anti rheumatic drug
csDMARDs: Conventional disease-modifying anti rheumatic drug
T2T: Treat-to-Target
METEOR: Measurement of Efficacy of Treatment in the ‘Era of Outcome’ in Rheumatology
LUMC: Leiden University Medical Center
VAS: Visual analogue scale
VASpt: Patients’ assessment of disease activity on a visual analogue scale
VASphys: Physicians’ assessment of patients’ disease activity on a visual analogue scale
ESR: Erythrocyte sedimentation rate
IQR: Interquartile ranges
GEE: Generalized estimating equations
SJC: Swollen joint counts
TJC: Tender joint counts
MTX: Methotrexate
EULAR: European League Against Rheumatism
CAMERA: Computer-Assisted Management in Early Rheumatoid Arthritis
DREAM: Dutch Rheumatoid Arthritis Monitoring
BeSt: Behandelstrategieën
TICORA: Tight Control in Rheumatoid Arthritis
ESPOIR: Etude et Suivi des POlyarthrites Indifférenciées Récentes
GUEPARD: Guérir la PolyArthrite
ACR: American College of Rheumatolgoy
