# Supplementary material for: DAS steered therapy in clinical practice; cross-sectional results from the METEOR database
Source: BMC Musculoskelet Disord. 2016 Jan 16;17:33. doi: 10.1186/s12891-016-0878-1 (PMC4715330; doi:10.1186/s12891-016-0878-1)
Supplement: Additional file 1: — Baseline characteristics (visit 1) for patients in the METEOR database. (DOCX 17 kb) [file 12891_2016_878_MOESM1_ESM.docx]

**Additional file 1: baseline characteristics (visit 1) for patients in the METEOR database**

**Format: Microsoft word**

**Table 1. Baseline characteristics (visit 1) for patients in the METEOR database ***

|  | **Total patients, n=1202** |
| --- | --- |
| Female, n (%) | 854 (71) |
| Age, mean (SD) | 56 (14) |
| Disease duration (mo), median (IQR) | 17 (3-84) |
| CCP, n (%) | 407 (71) |
| RF-Factor, n (%) | 722 (75) |
| DAS, mean (SD) | 2.5 (1.1) |
| HAQ, mean (SD) | 1.0 (0.7) |
| ESR, median (IQR) | 19 (9-34) |
| SJC, median (IQR) | 3 (0-7) |
| TJC, median (IQR) | 4 (1-7) |
| VAS, median (IQR)  Patient global  Doctor global  Patient pain | 39 (19-59)  21 (10-40)  43 (23-64) |

^*CRP=C-reactive Protein, ESR= Erythrocyte Sedimentation Rate, DAS28=Disease Activity Score 28 joints, HAQ= Health Assessment Questionnaire, IQR= Inter Quartile Range, CCP=Cyclic Cictrullinated Peptide Antibody, RF=Rheumatoid Factor^
